# Stable isotopic labelling of β-sitosteryl ferulate for use as analytical tool

**DOI:** 10.1016/j.fochx.2022.100227

**Published:** 2022-01-23

**Authors:** Sarah Mazzotta, Giovanna Baron, Laura Fumagalli

**Affiliations:** Department of Pharmaceutical Sciences, University of Milan, 20133 Milan, Italy

**Keywords:** γ-Oryzanol, Sitosteryl ferulate, Isotope labelling, Convenient synthesis, Rice bran

## Abstract

Rice is one of the major staple foods consumed worldwide and due to the presence of γ-oryzanol (γ-OZ) it is well-recognized as functional food. For this reason, the most appropriate varieties' identification in term of content of γ-OZ has become essential in order to assess their potential nutraceutical exploitation. This study reports a suitable and versatile procedure to obtain the isotopologues of sitosteryl ferulate, one of the major γ-OZ components, namely 3-O-(3-OC^2^H_3_-feruloyl)-β-sitosterol (**14**-*d*_3_) and 3-O-(3-OC^2^H_3_-8-^2^H-feruloyl)-β-sitosterol (**14**-*d*_4_) for use as analytical tool.

## Highlights

• Labelled sitosteryl ferulate, a component of γ-oryzanol mixture, has been synthesized for analytical applications.
• A methoxy-*d_3_* group was stably inserted on the aromatic ring of ferulic portion, together with a fourth deuterium incorporated on the double bond.
• The convenient synthesis could be applied for the preparation of other γ-oryzanol components.

## Introduction

About 2,500 years ago Hyppocrates, the father of medicine, paved the way for the “food as medicine” philosophy, by the statement “Let food be thy medicine and medicine be thy food”.

Over the years, the increase of knowledge in medicine field and the enhanced perception of the health care have led to refine Hyppocrates’ though establishing the concept of nutraceutical (da Costa, 2017). Indeed, many foods have been recognized both from a nutritional and pharmaceutical point of view.

As a consequence, we are now living in a new era in which the food industry has become a research-oriented sector.

**Fig. 1:**
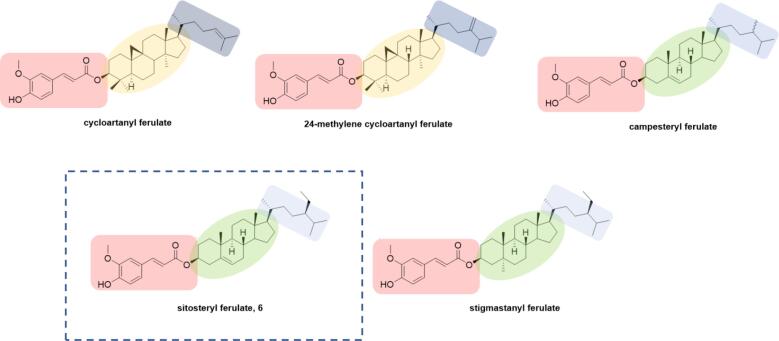
Main components of γ-oryzanol mixture from rice bran.

Among functional foods rice is well-recognized (Sawada et al., 2019, Sharif et al., 2014) because of γ-oryzanol (γ-OZ), which is a mixture of ferulic acid esters of triterpene alcohols and phytosterols considered nutriactive phytochemical naturally occurring in crude rice bran oil. The major components of γ-OZ mixture are cycloartanyl ferulate, 24-methylene cycloartanyl ferulate, campesteryl ferulate, sitosteryl ferulate and stigmastanyl ferulate (Fig. 1) (Angelis et al., 2011). Since rice is one of the major staple foods consumed worldwide and due to its numerous human health benefits, such as antioxidant, anti-inflammatory and anti-cancer activities, cholesterol lowering, prevention of obesity and diabetes, γ-OZ is of particular interest (Ghatak and S., & J. Panchal, S. , 2011, Berger et al., 2005, Szcześniak et al., 2016).

Furthermore, the content, the quality and the composition of phytochemicals in vegetables is greatly affected by plant genotype, harvest season, soil quality, and agronomic and environmental factors (Giovannetti et al., 2013). For these reasons, the most appropriate varieties' identification in term of content of γ-OZ has become essential in order to assess their potential nutraceutical exploitation (Castanho et al., 2019, Kim et al., 2015).

As general belief, to achieve reliable and accurate quantitative analysis stable isotopically labelled (SIL) internal standard is necessary.

In this work we report a suitable and versatile procedure to obtain the isotopologues of sitosteryl ferulate, one of the major γ-OZ components, namely 3-O-(3-OC^2^H_3_-feruloyl)-β-sitosterol (**14**-*d*_3_) and 3-O-(3-OC^2^H_3_-8-^2^H-feruloyl)-β-sitosterol (**14**-*d*_4_) for use as analytical tool. Additionally, we report also the synthesis of the not labelled sitosteryl ferulate (**6**) in order to immediately appreciate the incorporation of deuterium.

## Materials and methods

### General

All reagents were purchased from Merck KGaA, Darmstadt, Germany. NMR spectra have been recorded using Varian 300 Mercury spectrometer at 300 MHz for ^1^H NMR, and at 75.43 MHz for ^13^C NMR. The spin multiplicities are reported as s (singlet), d (doublet), t (triplet), q (quadruplet), m (multiplet), or br (broad signal). The high-resolution MS analysis was performed by direct infusion on a LTQ Orbitrap XL (Thermo Scientific, Bremen, Germany) equipped with an ESI source, operating in negative ion mode. The mass spectrometer acquired in full mass scan mode and resolution 30,000 FWHM at *m*/*z* 400.

### Synthesis of *trans*-4-O-acetylferulic acid (2)

To a solution of *trans*-ferulic acid (**1**, 2.60 mmol, 500.00 mg) in pyridine (7.00 mL), acetic anhydride (7.80 mmol, 0.73 mL) and a catalytic amount of 4-(dimethylamino)pyridine (10% mol, 30.16 mg) have been added. The reaction mixture has been stirred for 3 h at room temperature, then the reaction has been quenched by adding 30 mL of water and stirred for 30 min. The pH has been adjusted to 2 using 10% aqueous solution of HCl and the product has been extracted with ethyl acetate (2 × 30 mL). The combined organic layers have been dried over anhydrous Na_2_SO_4_, filtered and concentrated to dryness, to give compound **2** as yellow pale solid (83% yield). Rf = 0.66 (15:1 DCM/MeOH). mp 196–198 °C. ^1^H NMR data were in accordance with published literature values (Winkler-Moser et al., 2015) ^1^H NMR (CDCl_3_) *δ* 7.74 (d, *J* = 15.9 Hz, 1H), 7.19–7.05 (m, 3H), 6.40 (d, *J* = 16.0 Hz, 1H), 3.88 (s, 3H), 2.33 (s, 3H).

### Synthesis of 3-O-(*trans*-4-O-acetylferuloyl)-β-sitosterol (5)

Compound **2** (2.11 mmol, 500.00 mg) has been suspended in chloroform (5.00 mL) and a catalytic amount of dimethylformamide (DMF) (30% mmol, 0.05 mL), then thionyl chloride (2.53 mmol, 0.19 mL) has been added to the mixture. The reaction mixture has been refluxed for 4 h and checked through NMR. After the complete formation of the corresponding acyl chloride (**3**), anhydrous pyridine (3 mL) and β-Sitosterol (**4**, 2.11 mmol, 873.04 mg) have been added to the flask and the mixture has been stirred overnight at room temperature. The reaction has been quenched by the addition of water (3 mL) and diluted with chloroform (3 mL), then the phases have been separated and the organic layer has been washed with 10% aqueous solution of HCl (3 × 5 mL), 10% aqueous solution of NaHCO_3_ (5 mL) and brine (5 mL). The organic phase has been dried over anhydrous Na_2_SO_4_, filtered and concentrated under reduced pressure. The residue has been purified by flash chromatography using cyclohexane/ethyl acetate (9:1) as eluent, to give compound **5** as yellow pale solid (40% yield). R_f_ = 0.54 (cyclohexane/ethyl acetate 4:1); mp 172–174 °C. ^1^H NMR data were in accordance with published literature values (Winkler-Moser et al., 2015) ^1^H NMR (CDCl_3_) *δ* 7.62 (d, *J* = 16.0 Hz, 1H), 7.13 – 7.00 (m, 3H), 6.37 (d, *J* = 15.9 Hz, 1H), 5.41 (d, *J* = 5.2 Hz, 1H), 4.84 – 4.72 (m, 1H), 3.86 (s, 3H), 2.40 (d, *J* = 7.2 Hz, 1H), 2.32 (s, 3H), 2.09 – 1.78 (m, 5H), 1.71 – 0.78 (m, 38H), 0.69 (s, 3H).

### Synthesis of 3-O-(*trans*-feruloyl)-β-sitosterol (6)

To a solution of **5** (0.58 mmol, 370.02 mg) in chloroform/methanol (2:1, 18.00 mL), K_2_CO_3_ (20% mol, 16.59 mg) has been added and the reaction mixture was refluxed for 6 h. Then, the reaction has been quenched with saturated aqueous solution of NH_4_Cl (4 mL) and the organic layer has been washed with water (5 mL), dried over anhydrous Na_2_SO_4_ and filtered. The solvent has been removed under vacuum and crude product has been purified by crystallization from ethanol to give compound **6** as yellow pale solid (75% yield). R_f_ = 0.51 (cyclohexane/ethyl acetate 4:1); mp 134–135 °C. ^1^H and ^13^C NMR data were in accordance with published literature values (Winkler-Moser et al., 2015). ^1^H NMR (CDCl_3_) *δ* 7.60 (d, *J* = 15.9 Hz, 1H), 7.11 – 7.00 (m, 2H), 6.91 (d, *J* = 8.1 Hz, 1H), 6.27 (d, *J* = 15.9 Hz, 1H), 5.82 (br, 1H, D_2_O exchangeable), 5.40 (d, *J* = 4.8 Hz, 1H), 4.79 – 4.66 (m, 1H), 3.92 (s, 3H), 2.40 (d, *J* = 8.0 Hz, 1H), 2.04 – 1.78 (m, 5H), 1.73 – 0.76 (m, 38H), 0.69 (s, 3H). ^13^C NMR (CDCl_3_) *δ* 166.6, 147.8, 146.7, 144.5, 139.7, 127.1, 123.0, 122.7, 116.1, 114.7, 109.3, 73.8, 56.7, 56.0, 55.9, 50.0, 45.8, 42.3, 39.7, 38.3, 37.0, 36.6, 36.1, 33.9, 31.9, 29.1, 28.2, 27.9, 26.1, 24.3, 23.1, 21.0, 19.8, 19.3, 19.0, 18.77, 11.9, 11.8; HMRS (*m*/*z*) experimental [M - H]^-^ 589.4251 (delta ppm = -2.3)

### Synthesis of 3-bromo-4-hydroxybenzaldehyde (8)

4-Hydroxybenzaldehyde (**7**, 4.09 mmol, 500.00 mg) has been dissolved in DCM (5.00 mL) and H_2_O_2_ (2.05 mmol, 0.24 mL) and cooled to −5 °C. A solution of bromine (2.05 mmol, 0.11 mL) in DCM (4.00 mL) has been slowly added to the flask during 5 h and the resulting mixture was then stirred for 1 h at the same temperature. Afterwards, a 2% aqueous solution of NaHSO_3_ (4 mL) has been added at 0 °C and the reaction was stirred for 30 min at room temperature. The two phases have been separated and the inorganic layer has been extracted with DCM (3 × 4 mL). The organic layers have been dried over anhydrous Na_2_SO_4_, filtered and concentred to dryness. The crude product has been purified by flash chromatography using DCM as eluent to give compound **8** as white solid (75% yield) R_f_ = 0.42 (DCM); mp 121–123 °C. ^1^H NMR (CDCl_3_) *δ* 9.84 (s, 1H), 8.04 (s, 1H), 7.78 (d, *J* = 8.4 Hz, 1H), 7.15 (d, *J* = 8.4 Hz, 1H), 6.06 (br, 1H, D_2_O exchangeable).

### Synthesis of [3-OC^2^H_3_] vanillin (9-d_3_) and [3-OC^2^H_3_-7-^2^H] vanillin (9-d_4_)

To a suspension of compound **8** (2.00 mmol, 400.03 mg) in methanol‑*d_4_* (5.00 mL), previously prepared sodium methoxide-*d_3_* (6 mmol, 340.29 mg), CuI (2 mmol, 190.45 mg) and a catalytic amount of DMF (40% mol, 0.06 mL) have been added. The reaction mixture has been stirred in a sealed tube at 115 °C for 5 h, then it has been stirred for other 30 min in an open system at room temperature. The solvent has been removed under reduced pressure and the residue has been dissolved in *tert*-butyl methyl ether (3 mL); 10% aqueous solution of HCl was added, adjusting the pH to 2 and the two phases have been separated. The inorganic layer has been extracted with *tert*-butyl methyl ether (3 × 3 mL) and the combined organic phases have been dried over anhydrous Na_2_SO_4,_ filtered and evaporated under vacuum. The crude product has been further purified by flash chromatography using DCM as eluent to give compound **9** as yellow pale solid (80% yield). R_f_ = 0.70 (DCM/MeOH 98:2); mp 82–84 °C. ^1^H NMR (CDCl_3_) *δ* 9.83 (s, 1H), 7.49 – 7.36 (m, 2H), 7.04 (d, *J* = 8.5 Hz, 1H), 6.19 (br, 1H, D_2_O exchangeable). HMRS (*m*/*z*) experimental [M - H]^-^ 153.0537 (<1%, delta ppm = 3.1), 154.0592 (62%, delta ppm = 1.4), 155.0649 (37%, delta ppm = -1.9).

### Synthesis of [3-OC^2^H_3_] ferulic acid (10-d_3_) and [3-OC^2^H_3_-8-^2^H] ferulic acid (10-d_4_).

Malonic acid (1.42 mmol, 147.76 mg) has been suspended in toluene (2.00 mL) and trimethylamine (1.67 mmol, 0.23 mL). The mixture has been stirred for 5 min at room temperature, then compound **9** (1.29 mmol, 200.05 mg) and piperidine (17% mol, 0.02 mL) have been added to the flask and the reaction has been vigorously stirred at 60 °C for 6 h. Then, a 5% aqueous solution of NaHCO_3_ has been added and the biphasic system was mixed for 20 min. The phases have been separated and the aqueous one has been acidified to pH 2 with 10% aqueous solution of HCl. The resulting solid has been filtered and dried to give compound **10** as yellow pale solid, that has been used in the next step without further purification (86% yield). R_f_ = 0.36 (cyclohexane/ethyl acetate 1:1); mp 169–171 °C.^1^H NMR (CDCl_3_) *δ* 7.71 (d, *J* = 15.9 Hz, 1H), 7.11 (dd, *J* = 8.2 Hz, *J* = 1.8 Hz, 1H), 7.05 (d, *J* = 1.8 Hz, 1H), 6.93 (d, *J* = 8.2 Hz, 1H), 6.30 (m, 1H, merged signals of **10**-*d_3_* and **10**-*d_4_*, multiplicity derived from a doublet of **10**-*d_3_* and a broad signal of **10**-*d_4_*), 5.89 (br, 1H, D_2_O exchangeable).

### *Synthesis of (3-OC^2^H_3_-4-O-acetyl) ferulic acid (11-d_3_) and (3-OC^2^H_3_-8-^2^H-*4*-O-acetyl) ferulic acid (11-d_4_)*

Compound **11** has been prepared following the same procedure described for compound **2**. Yellow pale solid (86% yield). R_f_ = 0.64 (DCM/MeOH 15:1); mp 194–195 °C. ^1^H NMR (CDCl_3_) *δ* 7.74 (d, *J* = 15.9 Hz, 1H), 7.20 – 7.10 (m, 2H), 7.07 (d, *J* = 8.1 Hz, 1H), 6.40 (m, 1H, merged signals of **11**-*d_3_* and **11**-*d_4_*, multiplicity derived from a doublet of **11**-*d_3_* and a broad signal of **11**-*d_4_*), 2.33 (s, 3H).

### Synthesis of 3-O-(3-OC^2^H_3_-4-O-acetylferuloyl)-β-sitosterol (13-d_3_) and 3-O-(3-OC^2^H_3_-8-^2^H-4-O-acetylferuloyl)-β-sitosterol (13-d_4_)

Compound **13** has been prepared following the same procedure described for compound **5**. Yellow pale solid (30% yield). R_f_ = 0.55 (cyclohexane/ethyl acetate 4:1); mp 158–160 °C. ^1^H NMR (CDCl_3_) *δ* 7.62 (d, *J* = 15.9 Hz, 1H), 7.12–7-03 (m, 3H), 6.37 (m, 1H, merged signals of **13**-*d_3_* and **13**-*d_4_*, multiplicity derived from a doublet of **13**-*d_3_* and a broad signal of **13**-*d_4_*), 5.41 (d, *J* = 3.8 Hz, 1H), 4.83–4.67 (m, 1H), 2.41 (d, *J* = 7.9 Hz, 1H), 2.32 (s, 3H), 2.09 – 1.76 (m, 5H), 1.69 – 0.79 (m, 38H), 0.69 (s, 3H).

### Syntheisis of 3-O-(3-OC^2^H_3_-feruloyl)-β-sitosterol (14-d_3_) and 3-O-(3-OC^2^H_3_-8-^2^H-feruloyl)-β-sitosterol (14-d_4_)

Compound **14** has been prepared following the same procedure described for compound **6**. Yellow pale solid (90% yield). R_f_ = 0.50 (cyclohexane/ethyl acetate 4:1); mp 105–107 °C. ^1^H NMR (CDCl_3_) *δ* 7.60 (d, *J* = 15.9 Hz, 1H), 7.10 – 6.99 (m, 2H), 6.91 (d, *J* = 8.1 Hz, 1H), 6.27 (m, 1H, merged signals of **14**-*d_3_* and **14**-*d_4_*, multiplicity derived from a doublet of **14**-*d_3_* and a broad signal of **14**-*d_4_*), 5.83 (br, 1H, D_2_O exchangeable), 5.40 (d, *J* = 4.3 Hz, 1H), 4.83 – 4.67 (m, 1H), 2.40 (d, *J* = 7.8 Hz, 1H), 2.08 – 1.77 (m, 5H), 1.72 – 0.77 (m, 38H), 0.69 (s, 3H). ^13^C NMR (75 MHz, CDCl_3_) *δ* 166.6, 147.8, 146.7, 144.5, 139.7, 127.1, 123.0, 122.7, 116.1, 115.9, 114.6, 109.2, 73.9, 56.7, 56.2, 56.0, 50.0, 45.8, 42.3, 39.7, 38.3, 37.0, 36.6, 36.1, 35.5, 33.9, 31.9, 31.8, 29.6, 28.2, 27.9, 26.0, 24.3, 23.0, 21.0, 19.8, 19.3, 19.0, 18.8, 12.0, 11.9. HMRS (*m*/*z*) experimental [M - H]^-^ 591.4345 (<2%, delta ppm = -5.9), 592.4430 (62%, delta ppm = -2.0), 593.4476 (37%, delta ppm = -4.7).

## Results and discussion

Among the five major components of the γ-OZ mixture (Fig. 1), the sitosteryl ferulate (**6**) has been selected considering both the commercial availability and the cost of the sterol moiety.

**Scheme 1:**
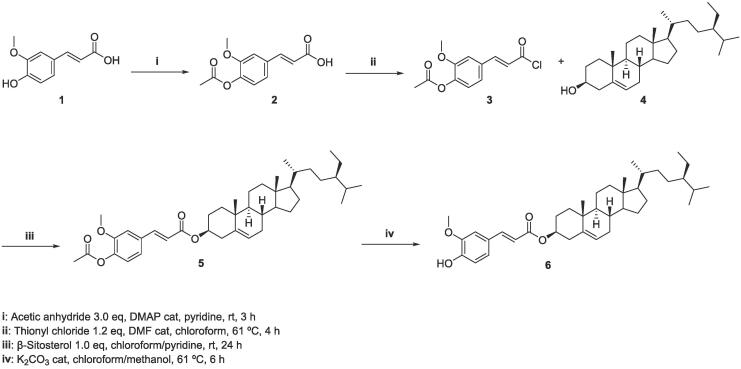
Synthetic route for 3-O-(*trans*-feruloyl)-β-sitosterol (**6**).

The synthesis of **6** has been performed following previously described procedures (Winkler-Moser et al., 2015) with some modifications. Commercially available *trans*-ferulic acid (**1**) has been employed as starting material, protecting the hydroxyl group in *para* position as acetyl ester (**2**), for the subsequent activation and coupling reaction with commercial β-sitosterol (**4**). Finally, the cleavage of acetyl group furnished the desired compound 3-O-(*trans*-feruloyl)-β-sitosterol **6** (Scheme 1).

**Scheme 2:**
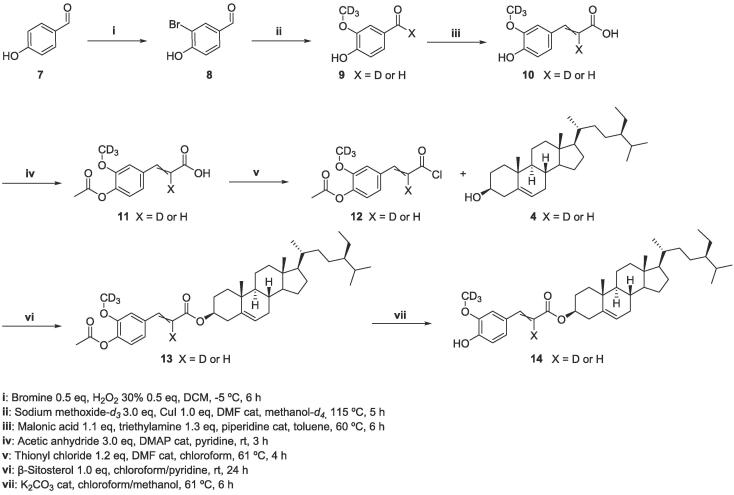
Synthetic route for 3-O-(3-OC^2^H_3_-feruloyl)-β-sitosterol (**14**-*d_3_*) and 3-O-(3-OC^2^H_3_-8-^2^H-feruloyl)-β-sitosterol (**14**-*d_4_*).

The preparation of the isotopic analogue of **6** has been planned in order to introduce a methoxy group (–OC^2^H_3_) in *meta* position of the aromatic ring. For this purpose, the starting material 4-hydroxybenzaldehyde **7** has been firstly mono-brominated, in order to provide a suitable substrate for the methoxylation reaction, using sodium methoxide-*d_3_,* that furnished the first labelled intermediate, [3-OC^2^H_3_] vanillin (**9**-*d_3_*). This reaction proceeded in good yield (80%) and represents a more efficient synthetic strategy to obtain **9**-*d_3_* with respect to previous described procedures (Gordon et al., 2013; S. W. Kim et al., 2009). The isotopic incorporation of *d_3_* reached the 100%. In addition, a further partial deuterium exchange was observed on the aldehyde carbonyl group (62% of the isotopologue **9**-*d_3_* and 37% of the isotopologue **9***-d_4_*) confirmed by HMRS, providing also [3-OC^2^H_3_-7-^2^H] vanillin (**9**-*d_4_*) (Scheme 2).

**Fig. 2:**
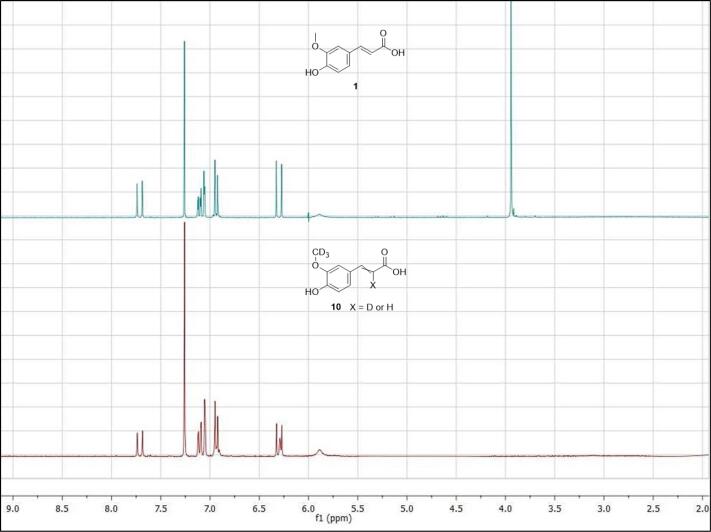
^1^H NMR spectra of commercial *trans*-ferulic acid (**1**) vs isotopologues [3-OC^2^H_3_] ferulic acid (**10**-*d*_3_) and [3-OC^2^H_3_-8-^2^H] ferulic acid (**10**-*d*_4_).

The subsequent step involved the Knoevenagel condensation to obtain [3-OC^2^H_3_] ferulic acid (**10**-*d_3_*) and [3-OC^2^H_3_-8-^2^H] ferulic acid (**10**-*d_4_*), in which the fourth deuterium is located on the double bond, confirmed by ^1^H NMR experiments. The Fig. 2 shows the ^1^H NMR spectra of *trans*-ferulic acid **1** and isotopologues **10**-*d_3_* and **10**-*d_4_*. From the spectra of **10** two differences can be observed with respect to the spectra of **1**: the former concerns the absence of the proton signal of the methoxy group at 3.94 ppm, and the latter is the appearance of a broad signal at 6.29 ppm. This signal shape is due to the deuterium insertion at the double bond, that couples to the vicinal proton with a very low coupling constant resulting in a merged signal with the doublet of the vicinal proton.

Once isotopically labelled ferulic acid has been prepared, the next steps involved the same synthetic procedures described above, furnishing the final desired compound, 3-O-(3-OC^2^H_3_-feruloyl)-β-sitosterol (**14**-*d_3_*) and 3-O-(3-OC^2^H_3_-8-^2^H-feruloyl)-β-sitosterol (**14**-*d_4_*) (Scheme 2). The incorporation of three and four deuteriums has been confirmed by ^1^H NMR and HMRS experiments (62% of the isotopologue **14**-*d_3_* and 37% of the isotopologue **14***-d_4_*).

## Conclusions

In conclusion, this work reports a convenient synthesis to prepare deuterium labelled sitosteryl ferulate (**14**), one of the main components of γ-OZ mixture. The procedure allowed to obtain the 100% of isotopic incorporation [*d_3_*] and also a partial (37%) introduction of another deuterium ([*d_4_*] isotopologue), confirmed by ^1^H NMR and HMRS analysis. This result represents the first report of deuterium labelling of γ-OZ, which can be considered a useful tool for the identification and quantification of these compounds from different varieties of rice bran. Moreover, applying the same synthetic strategy, other isotopically labelled γ-OZ components can be obtain through the substitution of the sitosterol moiety.

## Funding

This research is part of the project “MIND FoodS HUB (Milano Innovation District Food System Hub): Innovative concept for the eco-intensification of agricultural production and for the promotion of dietary patterns for human health and longevity through the creation in MIND of a digital Food System Hub”, cofunded by POR FESR 2014-2020_BANDO Call HUB Ricerca e Innovazione, Regione Lombardia.

## Declaration of Competing Interest

The authors declare that they have no known competing financial interests or personal relationships that could have appeared to influence the work reported in this paper.
