# Supplementary material for: Stable isotopic labelling of β-sitosteryl ferulate for use as analytical tool
Source: Food Chem X. 2022 Jan 23;13:100227. doi: 10.1016/j.fochx.2022.100227 (PMC9039913; doi:10.1016/j.fochx.2022.100227)
Supplement: Supplementary data 1 [file mmc1.docx]

**Supplementary material**

**Stable isotopic labelling of β-sitosteryl ferulate for use as analytical tool.**

Sarah Mazzotta, Giovanna Baron, Laura Fumagalli*

Department of Pharmaceutical Sciences, University of Milan, 20133 Milan, Italy.

*Corresponding author’s e-mail: laura.fumagalli@unimi.it

CONTENTS

1. **Figures S2-S6.** NMR spectra of compounds **6**, **9** and **14**.
2. **Figure S7.** HMRS spectra of compounds **6**, **9**, and **14**.

**Figure S2.** ^1^H NMR spectra of 3-O-(*trans*-feruloyl)-β-sitosterol (**6**).


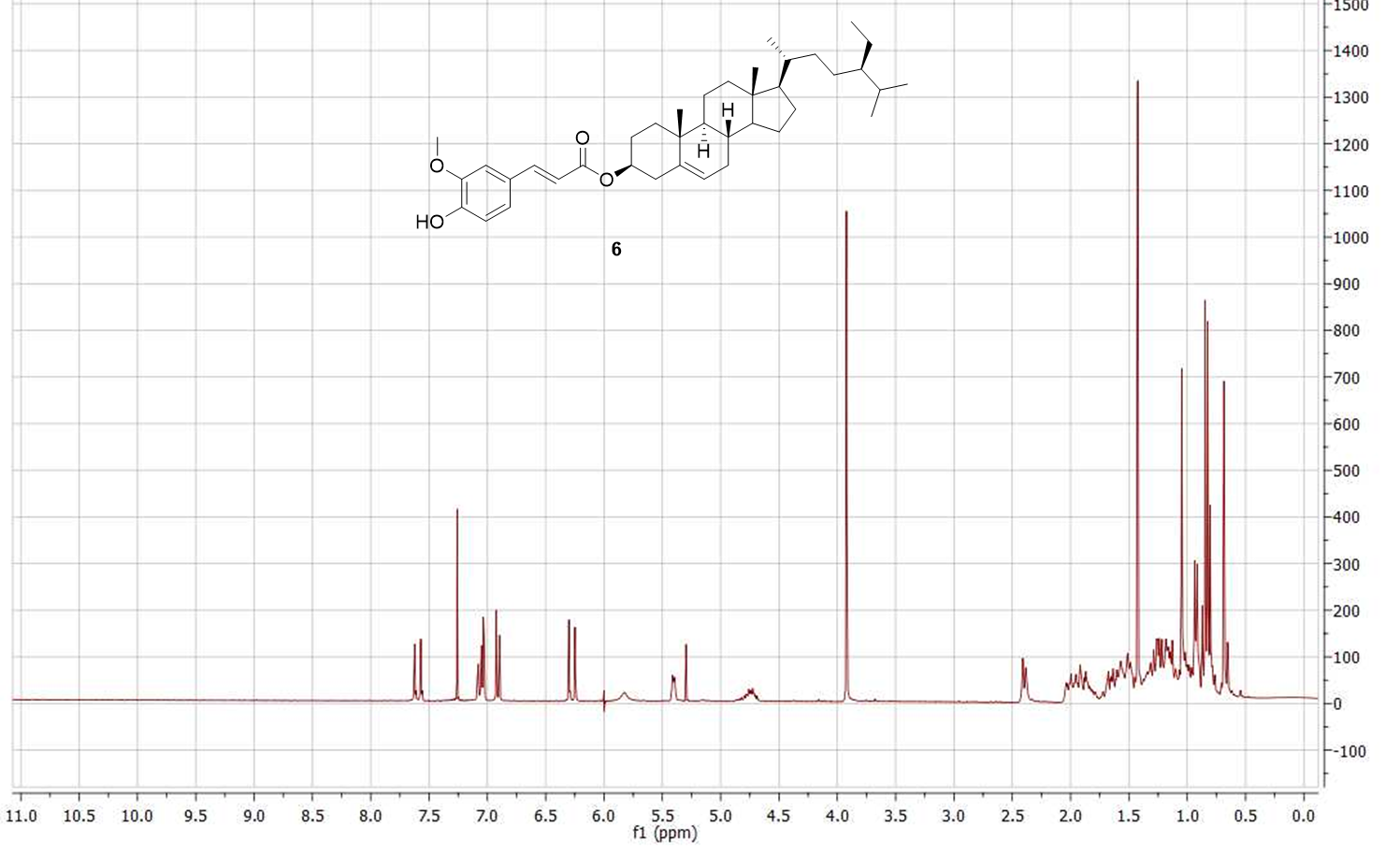


**Figure S3.** ^13^C NMR spectra of 3-O-(*trans*-feruloyl)-β-sitosterol (**6**).


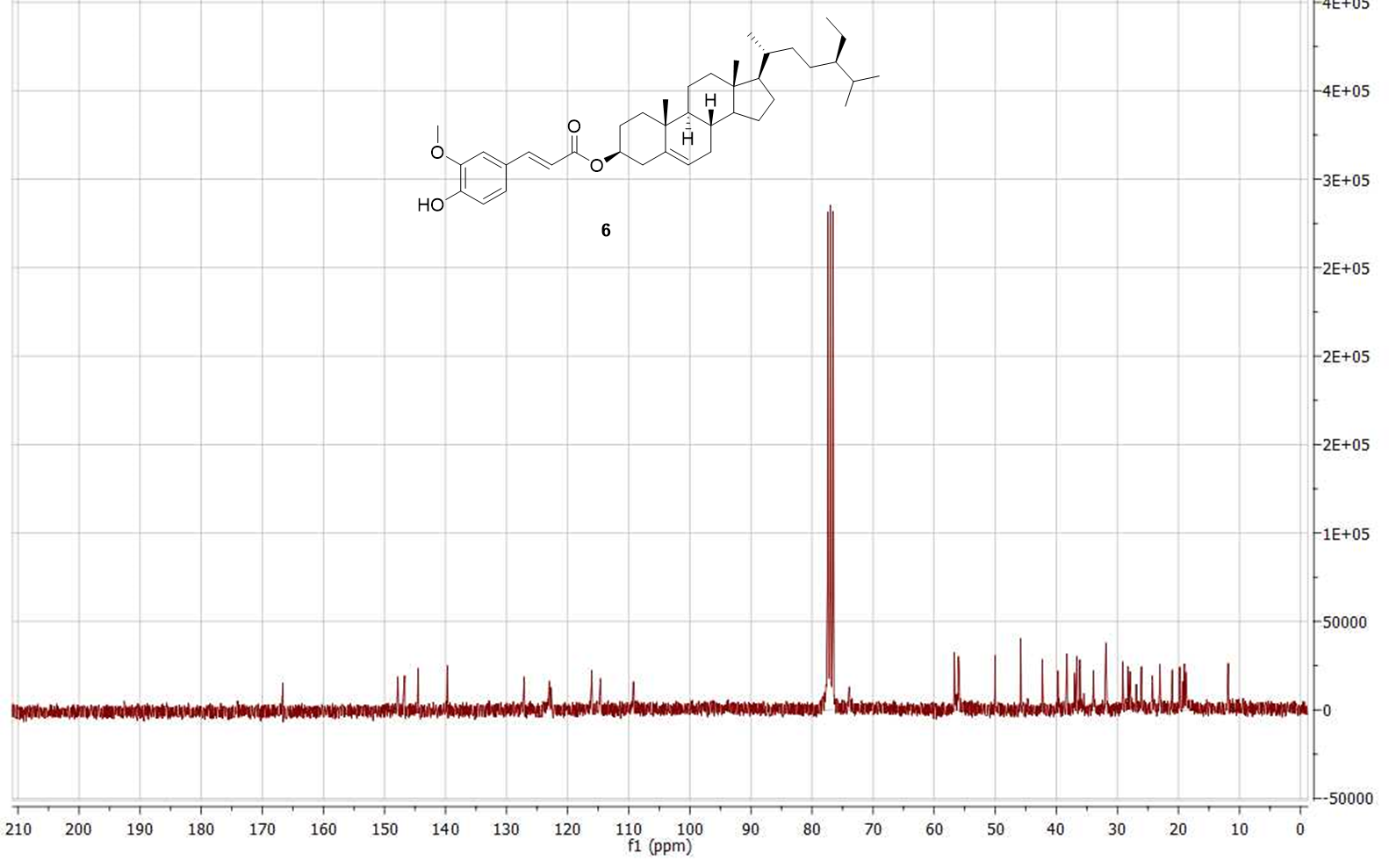


**Figure S4.** ^1^H NMR spectra of [3-OC2H3] vanillin (**9**-*d*_3_) and [3-OC2H3-7-2H] vanillin (**9**-*d*_4_).

**
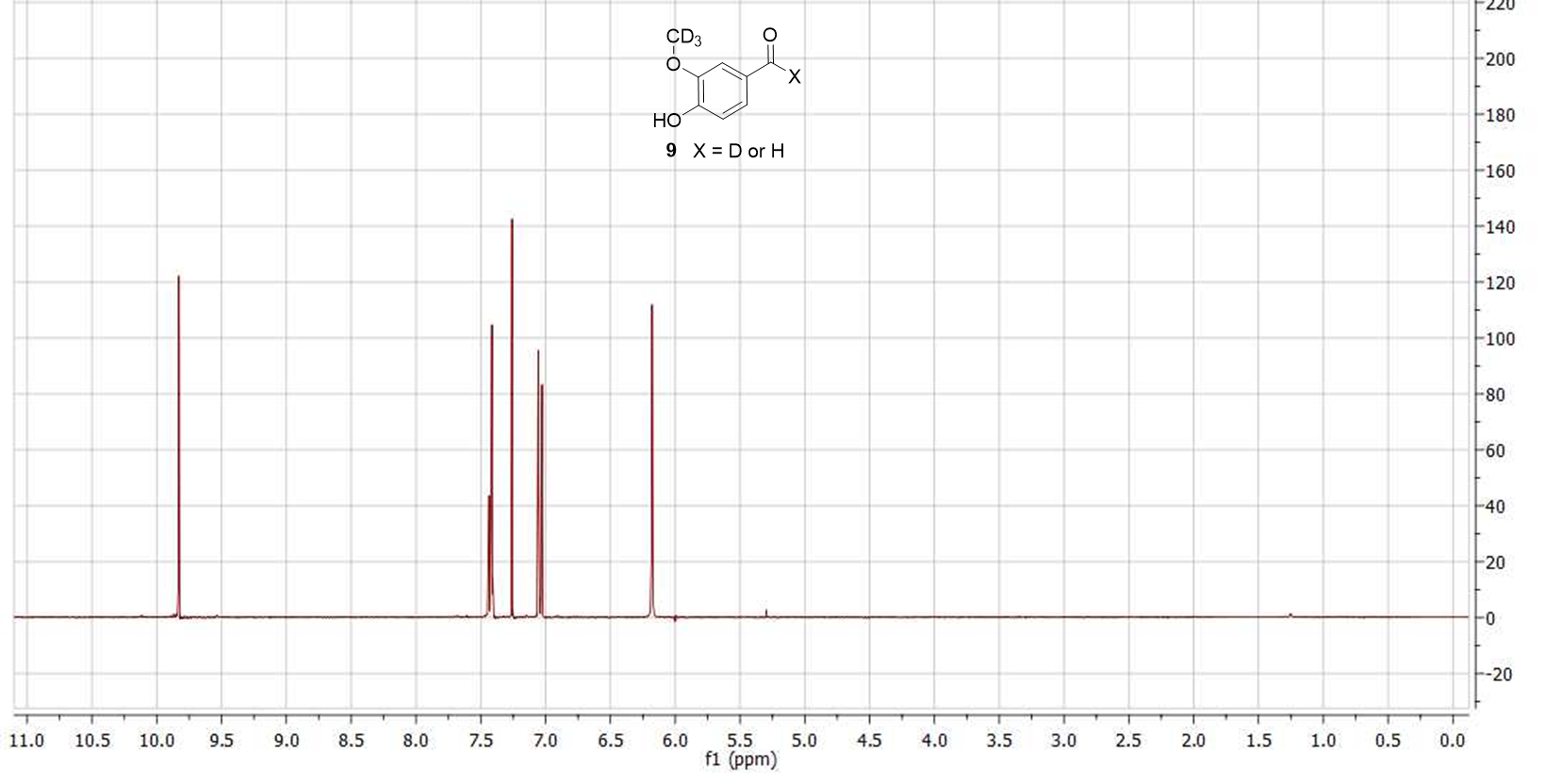
**

**Figure S5.** ^1^H NMR spectra of 3-O-(3-OC^2^H_3_-feruloyl)-β-sitosterol (**14**-*d_3_*) and 3-O-(3-OC^2^H_3_-8-^2^H-feruloyl)-β-sitosterol (**14**-*d_4_*).

*
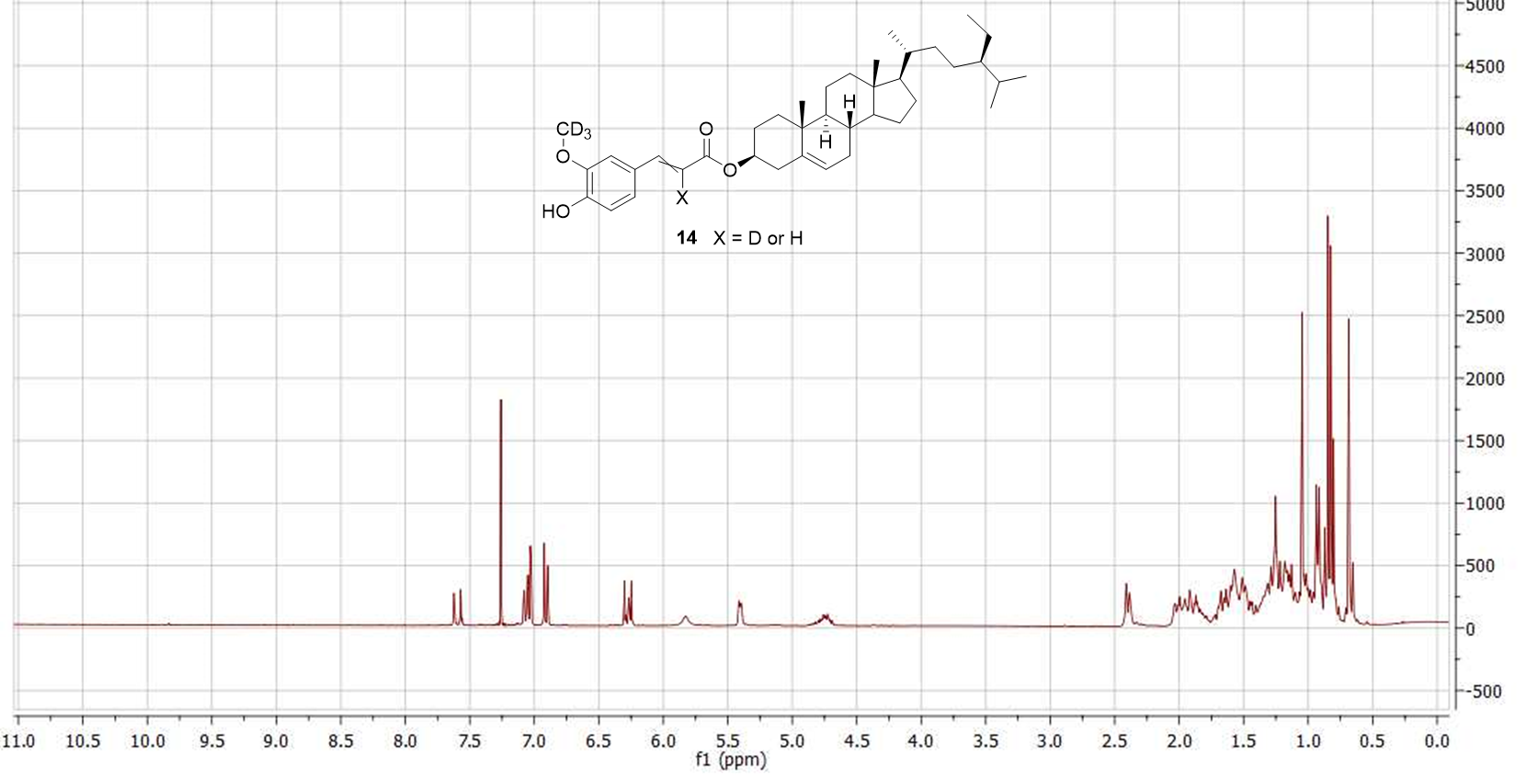
*

**Figure S6.** ^13^C NMR spectra of 3-O-(3-OC^2^H_3_-feruloyl)-β-sitosterol (**14**-*d_3_*) and 3-O-(3-OC^2^H_3_-8-^2^H-feruloyl)-β-sitosterol (**14**-*d_4_*).

**
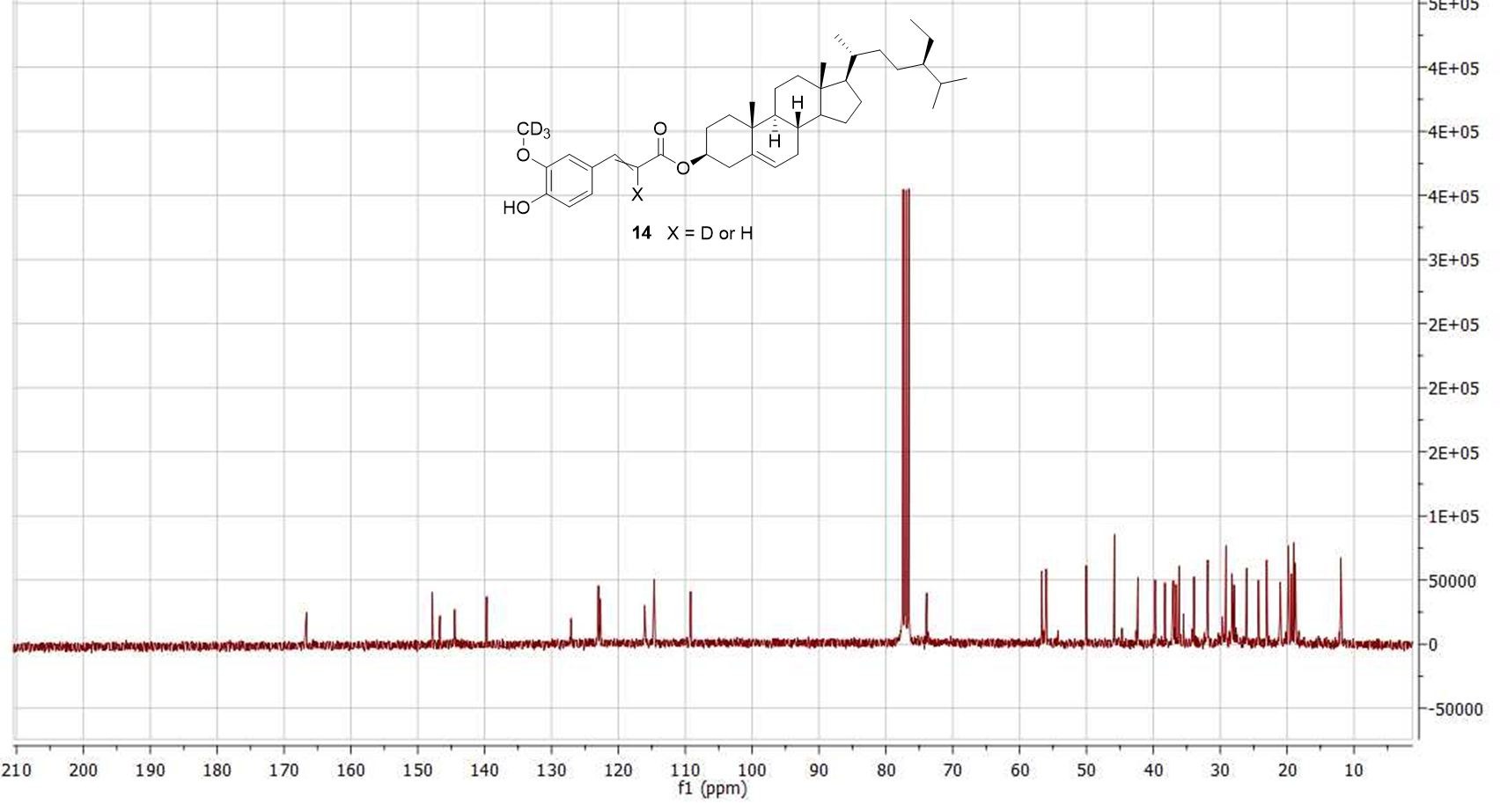
**

**Figure S7.** HMRS spectra of compounds **6**, **9** and **14**.

**
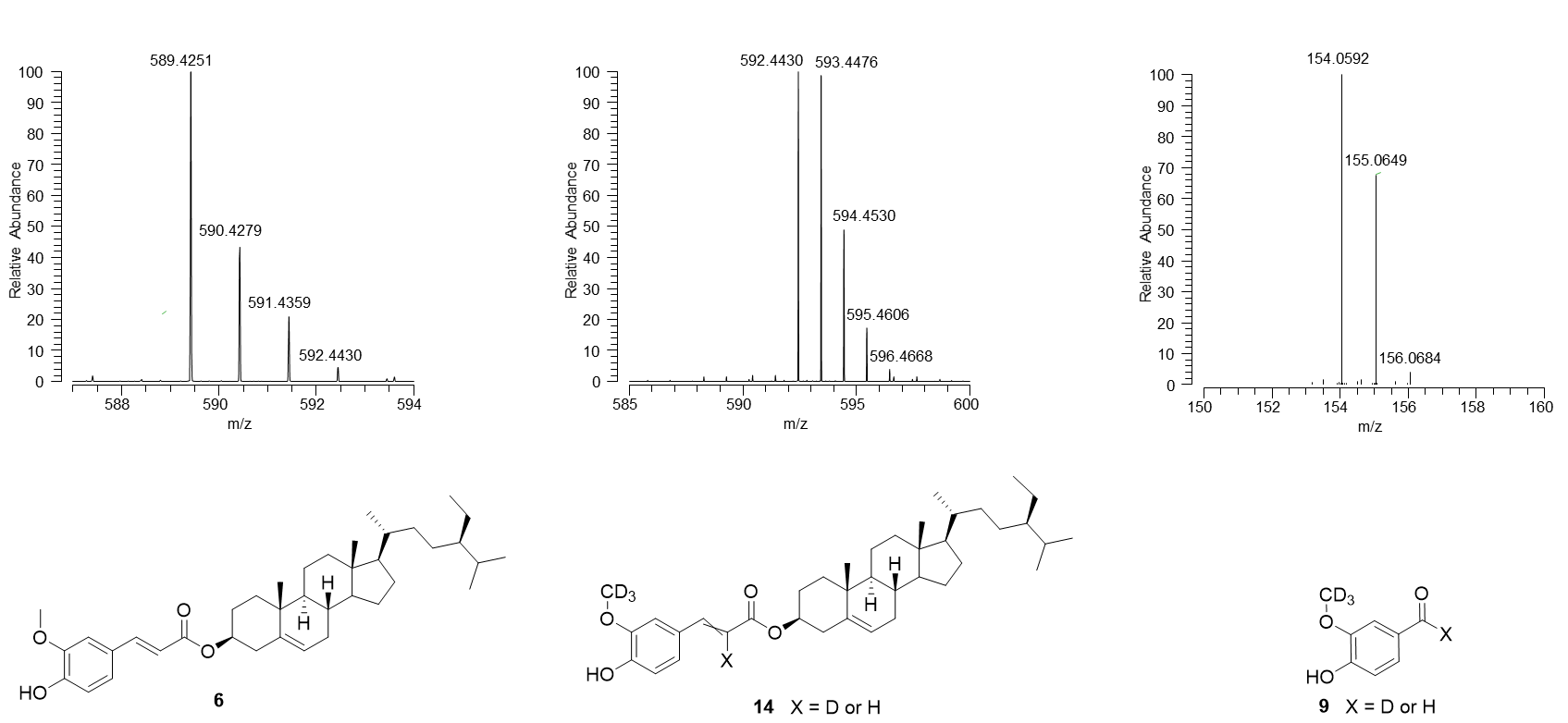
**
